# The impact of technical failures on recombinant production of soluble proteins in *Escherichia coli*: a case study on process and protein robustness

**DOI:** 10.1007/s00449-021-02514-w

**Published:** 2021-01-24

**Authors:** Alexander Pekarsky, Melanie Reininger, Oliver Spadiut

**Affiliations:** Institute of Chemical, Environmental and Bioscience Engineering, Research Area Biochemical Engineering, Technische Universität Wien, TU Wien, Gumpendorferstrasse 1a, 1060 Vienna, Austria

**Keywords:** Process deviation, *Escherichia coli*, Cytosolic protein, Robustness, Bioreactor, Protein glycation

## Abstract

Technical failures lead to deviations in process parameters that can exceed studied process boundaries. The impact on cell and target protein is often unknown. However, investigations on common technical failures might yield interesting insights into process and protein robustness. Recently, we published a study on the impact of technical failures on an inclusion body process that showed high robustness due to the inherent stability of IBs. In this follow-up study, we investigated the influence of technical failures during production of two soluble, cytosolic proteins in *E. coli* BL21(DE3). Cell physiology, productivity and protein quality were analyzed, after technical failures in aeration, substrate supply, temperature and pH control had been triggered. In most cases, cell physiology and productivity recovered during a subsequent regeneration phase. However, our results highlight that some technical failures lead to persistent deviations and affect the quality of purified protein.

**Supplementary Information:**

The online version contains supplementary material available at 10.1007/s00449-021-02514-w.

## Introduction

Process and product quality robustness are of utmost importance for the industry. In bioprocess development, a protein production process is usually characterized in a broad range of different process conditions (design space) and later optimized in a narrow range (control space). This can be done using a design of experiments (DoE) approach that statistically investigates how a system output variable (e.g. protein titer) changes upon the variation of a certain input variable (e.g. temperature) or a variety of input variables [1]. This approach does not only allow process development and optimization, but also robustness testing [1, 2].

In contrast to most lab-scale bioprocesses, pilot and large-scale bioprocesses often apply fully digitized bioprocess monitoring and control systems. Nevertheless, unexpected equipment failures and operator errors might cause process deviations (e.g., a failure in the cooling system might cause an increase in temperature). Knowledge on the effects of these technical failures on cell and product are scarce. Recently, we have shown that an inclusion body (IB) process with *E. coli* BL21(DE3) is robust to temporary technical failures during the protein production phase [3]. In this follow-up study, we want to know how soluble protein production in the cytosol and protein quality are affected by such technical failures. In Table 1 we give an overview of the technical failures that we investigated and our hypotheses, how this might affect the bioprocess and the product [3].

**Table 1:** Impact of technical failures on a bioprocess and the quality and quantity of recombinant, soluble, cytosolic protein

		Impact on bioprocess parameter				
		Dissolved oxygen (DO)	Temperature	pH	N-source	C-source
Technical failure	Interruption of aeration	DOWN				
	Interruption of feeding	UP				DOWN
	Failure in pH control			DOWN or UP	DOWN or UP^a^	
	Failure in T control		UP			
	Overfeeding					UP

		Impact on soluble protein			
		IB formation	Soluble protein productivity	Degradation	Protein homogeneity (Isoelectric point; Hydrophobicity; Size)
Process scenario	DO or OTR^b^ decrease		YES [4]		YES
	Steep T increase	YES [5]	YES [5, 6]	YES [6]	YES
	Steep pH increase/decrease	YES [5]	YES [5, 7]		YES
	Substrate accumulation	YES [5]	YES [5]		YES
	Substrate starvation		YES		YES

Data were collected by literature review and lab experience. Upper part shows impact of technical failures during the upstream process on bioprocess parameters. Lower part shows impact of resulting scenarios on cell physiology as well as soluble protein quality and quantity

^a^If the base is also a nitrogen source (e.g. NH_4_OH)

^b^OTR…oxygen transfer rate

With respect to protein quality, *E.* coli has rather simple protein modification capabilities, like enzyme cofactor integration [8] or disulphide bridge formation in its periplasm [9]. However, spontaneous protein glycation has been reported [10–12]. Protein glycation is an unwanted, covalent, non-enzymatic modification that changes the physico-chemical properties of a protein and increases its size by 178 Da and/or 258 Da [11]. The sugar intermediate 6-phosphogluconolactone (6-PGL) of the pentose phosphate pathway (PPP) has been identified as the main substrate for protein glycation (gluconoylation) [11, 13]. Especially proteins with N-terminal hexa-histidine (His_6_) tag are gluconoylated [11, 14]. More importantly, the highly used *E. coli* BL21 strain accumulates 6-PGL under aerobic conditions [15]. Thus, protein glycation has to be considered, especially for recombinant protein production in *E. coli* BL21 strains.

In this follow-up study [3], we investigated the impact of bioreactor-related technical failures during recombinant protein production on (1) cell physiology and productivity of *E. coli* BL21(DE3) and (2) the quality of two recombinant, cytosolic model proteins. The recombinant proteins were chosen to generate results for (1) proteins with a simple fold that are easily expressed to high soluble titers (like green fluorescence protein plus (GFP) [16]); and for (2) proteins with a complex fold that are prone to form intracellular IBs upon overexpression, and thus have to be expressed at low growth rates (like pyranose-2-oxidase (P2Ox) [8]).

## Materials and methods

### Chemicals

All chemicals were purchased from Carl Roth GmbH (Vienna, Austria), if not stated otherwise.

### Strains and recombinant proteins

For all experiments, an *E. coli* strain BL21(DE3) (Genotype: *E. coli* str. B F^–^ ompT gal dcm lon hsdS_B_(r_B_^–^m_B_^–^) λ(DE3 [lacI lacUV5-T7p07 ind1 sam7 nin5]) [malB^+^]_K-12_(λ^S^)), transformed with a pET21d( +) vector carrying the codon-optimized gene for green fluorescent protein plus (GFP) [16] or pyranose-2-oxidase (P2Ox) [8] was used for cytosolic expression. Both recombinant strains had been generated in-house in previous studies. Recombinant proteins differed in size, isoelectric point and folding (Table 2).

**Table 2:** Overview of the recombinant model proteins

	GFP	P2Ox
Molecular weight with His_6_ [17]	~ 28.1 kDa (monomeric)	~ 280 kDa (homotetrameric)
Isoelectric point with His_6_ [17]	~ 6.13	~ 5.69
Cofactors	–	1 × FAD per monomer
Purification-tag	His_6_ (C-terminus)	His_6_ (C-terminus)
Activity mesaurement	Fluorescence at 512 nm (excitation at 491 nm)	Oxidation of glucose
Reference for	Proteins with simple fold	Proteins with complex fold
Protein described in	[16]	[8]

### Conditions for soluble recombinant protein production

Process conditions to reduce IB formation and allow the formation of soluble protein were adjusted based on our own experiences and literature [18]. Soluble GFP production was done at 27.5 °C, pH 7.0 and induced with 0.5 mM isopropyl β-D-1-thiogalactopyranoside (IPTG). Soluble P2Ox production was screened at 20 °C, 25 °C and 30 °C, and at pH 6.8, pH 7.0 and pH 7.2 and finally with 0.5 mM and 1 mM IPTG in shake flask cultivations with DeLisa medium [19]. Highest soluble, cytosolic P2Ox production was reached with 0.5 mM IPTG, 20.0 °C and pH 7.2 (data not shown).

### Upstream process

A total of 8 bioreactor cultivations was performed for each recombinant *E. coli* BL21(DE3) strain. The batch was inoculated with pre-cultured cells from shake flasks. Each cultivation consisted of a batch phase for biomass generation, a controlled exponential fed-batch to around 30 g L^−1^ dry cell weight (DCW), and an IPTG induced protein production phase. Process raw data can be found in Supplementary Figure. 1 to 16.

#### Media composition

The preculture medium was prepared according to DeLisa et al*.* [19], but with additional 0.1 g L^−1^ ampicillin as antibiotic selection agent. The batch medium contained the same components as the preculture medium, but with 22.0 g L^−1^ D( +)-glucose monohydrate, 1 g L^−1^ Antifoam PPG 2000 (SigmaAldrich, Austria) and without antibiotic agents. Use of antibiotic agents above shake flask scale is usually not employed in the industry when possible. Feed medium contained per litre: 445 g D ( +) glucose monohydrate, 20.23 g MgSO_4_ × 7H_2_O, 0.04045 g Fe(III)citrate, 0.01315 g EDTA, 0.01618 g Zn(CH_3_COO)_2_ × 2 H_2_O, and 8.09 ml trace element solution.

#### Preculture and batch phase

Preculture and batch cultivations in the bioreactor were performed analogous to our previous study [3].

#### Fed‑batch and induction phase

After the batch phase, the exponential fed-batch phase was initiated. The temperature was controlled at 37 °C, the DO above 30% and the pH to pH 7.0 for GFP or pH 7.2 for P2Ox. The feed was added at a specific glucose uptake rate (q_S, Glc_) of around 0.25 g g^−1^ h^−1^ with a biomass yield (Y_X/S_) of 0.44 g g^−1^ (i.e. a specific growth rate (µ) = 0.11 h^−1^).

Prior to induction, the temperature was set to 27.5 °C (GFP cultivations) or 20.0 °C (P2Ox cultivations) to reduce cellular stress and IB formation. Further, IB formation was reduced through a switch from exponential to constant linear feeding (µ decreases over time), which is also common in industrial processes [20]. Recent data on recombinant protein production with *E. coli* BL21(DE3) also showed that exponential feeding, depending on induction duration and target µ, lead to increased cell death and substrate accumulation in the medium over time [21]. Therefore, we chose a combination of low µ, low temperature and prolonged induction duration to reduce stress (and IB formation). For recombinant GFP production, we chose an initial q_S, Glc_ of 0.214 g g^−1^ h^−1^ with a Y_X/S_ of 0.35 g g^−1^ (µ = 0.075 h^−1^). P2Ox is a large multimeric enzyme, thus formation of IBs is more likely [22]. Therefore, we decreased feeding by approximately 50% compared to GFP to an initial q_S, Glc_ of 0.100 g g^−1^ h^−1^ with a Y_X/S_ of 0.35 g g^−1^ (µ = 0.035 h^−1^). A single IPTG pulse to 0.5 mM was added to initiate induction. The total induction time was 12 h.

#### Introduction of technical failure

Technical failures were triggered based on communication with industrial partners and analogous to our previous study ([3]; Table 3).

**Table 3:** Cultivations with and without technical failures

Cultivation	Technical failure	Theoretical origin	Real origin
C1–C3	Reference runs		
C4	Interruption of aeration	e.g., Blocked inlet filter	Aeration turned off
C5	Interruption of feeding	e.g., Empty feed tank	Feed pump stopped
C6	Failure in T control	e.g., Heat exchanger defect	T control turned off
C7	Overfeeding	e.g., Pump communication problem	Set pump to maximum flow rate
C8	Failure in pH control	e.g., Base pump defect	pH control turned off

After introduction of the technical failure, a subsequent deviation phase of 1.5 h followed. This duration mimicked the estimated time from detection of the technical failure to its repair [3]. Subsequently, cultures were run for another 4.5 h under previous process conditions (regeneration phase) to yield a total of 12 h induction time.

#### Sampling strategy

Samples were taken at the beginning of the fed-batch; start of induction, start of deviation phase; end of deviation phase; and at the end of the regeneration phase (end of fermentation). An aliquot of at least 50 ml was centrifuged and the biomass was snap-frozen in liquid N_2_ for later use.

#### Sample analysis

The DCW concentration was determined in triplicates by centrifugation of 5 ml culture broth (4000 g, 4 °C, 10 min), washing the pellet once with 5 ml 0.9% NaCl, subsequent drying for 72 h at 105 °C and weighing. Optical density of the culture broth was measured in duplicates at 600 nm (OD_600_) using a spectrophotometer (Genesys 20; ThermoFisher Scientific, Vienna, Austria). Protein concentration of cell-free supernatant was determined at 595 nm using the Bradford Protein Assay Kit (Bio-Rad Laboratories GmbH, Vienna, Austria) with bovine serum albumin (BSA) (protein standard; micro standard, liquid; P0914; SigmaAldrich, Austria) as standard. Lysis was investigated by extracellular double-stranded DNA (dsDNA) using the PicoGreen assay kit (ThermoFisher Scientific, Austria). Concentration of glucose and other metabolites was determined in cell-free samples by high-pressure liquid chromatography (HPLC) measurement (U3000, ThermoFisher Scientific, USA) equipped with a Supelco guard column and a Supelco gel C-610H ion-exchange column (SigmaAldrich, Austria) and a refractive index detector (Agilent Technologies, USA). The mobile phase was 0.1% H_3_PO_4_ with a constant flow rate of 0.5 ml min^−1^ and the system was run isocratically. Calibration was done by measuring standard points in the range of 0.1–10 g L^−1^. Along with the observed standard deviations for the measurements of DCW, glucose and metabolite concentrations, the errors were propagated to the specific rates as well as to the yield coefficients.

#### Soluble protein analysis

Intracellular target protein analysis was done after high-pressure homogenization (HPH) of the frozen biomass aliquots. HPH was performed with a PandaPLUS 2000 (GEA Mechanical Equipment, Italy). The biomass was re-suspended in IMAC buffer A (100 mM TRIS; 500 mM NaCl; 20 mM Imidazole; 8% v/v Glycerol; pH 7.5) to 15 g L^−1^ DCW and processed at 1000 bar for 3 passages. After centrifugation, the supernatant was used for soluble protein analysis; the remaining pellet was frozen and later used for IB analysis. Target protein concentration was determined by reversed-phase (RP) HPLC (BioResolve RP mAb Polyphenyl, 450 Angstrom, 2.7 µm, 4.6 × 100 µm from Waters GmbH, Austria) with a linearly increasing gradient of acetonitrile (AppliChem, Germany) with 0.1% trifluoroacetic acid (SigmaAldrich, Austria), a flowrate of 1.2 ml min^−1^ and at 75 °C. Target protein standards from shake flasks (see below) were used for HPLC quantification of IBs and the soluble target protein.

For the determination of P2Ox activity, a 2,2′-azino-bis(3-ethylbenzothiazoline-6-sulfonic acid) (ABTS) assay was used. The reaction solution included 14.7 mg ABTS, 100 µl peroxidase solution (1.5 mg horseradish peroxidase (SigmaAldrich, Austria) in 1 ml 50 mM TRIS; pH 7.5; 1 M (NH_4_)_2_SO_4_) filled up to 2.5 ml with 50 mM TRIS buffer, pH 7.5. The reaction mixture was then composed of 20 µl reaction solution, 12 µl 0.5 M d-glucose in 50 mM TRIS buffer, pH 7.5 and 164 µl 50 mM TRIS buffer, pH 7.5. After incubation at 30 °C for 5 min, 4 µl of the cell-free sample were added and increase in absorption at 420 nm was monitored over 3 min with a plate reader (Infinite M200 pro; Tecan Austria GmbH, Austria). The measurement was performed in triplicates and the volumetric activity was calculated with Eq. 1.

1 $${\text{Volumetric activity}} \left[ {\frac{{\text{U}}}{{{\text{mL}}}}} \right] = \frac{{{\text{V}}_{{{\text{total}}}} *\frac{{\Delta {\text{Abs}}}}{\min }*{\text{dilution}}}}{{{\text{V}}_{{{\text{sample}}}} * \varepsilon *d}}$$

V_total_—total volume in cuvette in μL; ∆Abs min^−1^—slope in absorption per min; dilution—potential dilution of the sample; V_sample_—volume of sample in cuvette in μL; ε—extinction coefficient (ε_420_ = 43.2 mM^−1^ cm^−1^); d—pathlength.

#### IB analysis

The cell debris was washed twice with deionized water. Afterwards, the pellet was weighed and diluted to a concentration of 100 g L^−1^ wet weight with solubilization buffer (7.5 M Guanidine-HCl, 62 mM TRIS, 100 mM dithiothreitol (SigmaAldrich, Austria); pH 7.5) and incubated on a shaker at RT for 40 min. After centrifugation, the supernatant was used for RP HPLC analysis to determine IB concentration.

### Downstream process

After HPH of the cultivation end biomass and centrifugation, the supernatant was directly used for protein purification by immobilized metal affinity chromatography (IMAC). An ÄKTA pure (GE Healthcare, Austria), equipped with a 1 ml HisTrapFF IMAC column (GE Healthcare, Austria) was used. The column was equilibrated with 10 column volumes (CV) IMAC buffer A. Subsequently, the column was loaded approximately 50% exceeding its dynamic binding capacity. Overloading was performed to saturate the column with his-tagged target protein to reduce adsorption of host cell proteins. Elution was performed with a linear gradient to 100% IMAC buffer B (100 mM TRIS; 500 mM NaCl; 500 mM Imidazole; 8% (v/v) Glycerol; pH 7.5) over 10 CV with a flow rate of 156 cm h^−1^. Flowthrough and elution fractions with specific target protein absorption were pooled. Specific absorption for GFP and P2Ox was measured at 491 nm and 395 nm, respectively.

#### Generation of target protein standards

Protein standards for HPLC analytics were generated in shake flask cultures similarly to preculture cultivations but with the addition of 0.5 mM sterile IPTG at 27.5 °C (GFP) or 20.0 °C (P2Ox). After 24 h, the broths were centrifuged, the biomass was re-suspended in IMAC buffer A and homogenized. After IMAC purification, active fractions were pooled and desalted with 20 mM TRIS, pH 8.4, 8% (v/v) glycerol using PD-10 columns (GE Healthcare, Austria). Protein concentration of the final, desalted pool was determined by bicinchonic acid assay (BCA) at 562 nm.

#### SDS-PAGE and protein glycation analysis

Sodium dodecyl sulphate polyacrylamide gel electrophoresis (SDS-PAGE) analysis was performed with 4–15% gradient gels (Mini-PROTEAN TGX Stain-Free Gels; Bio-Rad Laboratories; USA). SDS-PAGE protein glycation analysis was performed with self-casted gels in a gel casting cassette (height: 100 mm; width: 100 mm; thickness: 0.75 mm; Bio-Rad Laboratories; USA). An 8% resolving gel and a 4% stacking gel were prepared the following: per ml resolving gel, we added 0.2 ml of 40% stock solution of 29:1 acrylamide:bisacrylamide (Fisher Scientific, Austria), 0.25 ml of 1.5 M TRIS; pH 8.8, 10 µl of 10% SDS solution, 0.535 ml of deionized H_2_O, 0.5 µl of TEMED (SigmaAldrich, Austria) and 5 µl of a 10% ammonium sulphate (APS) solution (SigmaAldrich, Austria). Per ml stacking gel we added 0.1 ml of 40% stock solution of 29:1 acrylamide:bisacrylamide (Fisher Scientific, Austria), 0.252 ml of 0.5 M TRIS; pH 6.8, 10 µl of 10% SDS solution, 0.636 ml of deionized H_2_O, 1 µl of TEMED (Sigma-Aldrich, Austria) and 5 µl of a 10% APS solution (SigmaAldrich, Austria). For increased retention of protein glycation products [23], we dissolved a respective amount of 3-(methacryloylamino)phenylboronic acid MPBA 0.13% (w/v) (Sigma-Aldrich, USA) in the resolving gel solution before polymerization was initiated with TEMED and APS. This led to increased retention of glycated protein species in SDS-PAGE analysis. All gels were run with 1 × SDS-PAGE running buffer (3 g L^−1^ TRIS, 14 g L^−1^ glycine, 1 g L^−1^ SDS. All samples were prepared with 4 × denaturation buffer (1 L contained 250 ml of 1 M TRIS, pH 6.8; 100 g SDS; 500 ml pure glycerol, 2.5 g bromophenol blue and 5 ml 2-mercaptoethanol) and heated at 95 °C for 10 min before use. The 4–15% gradient gels were run at 160 V for 40 min with the Precision Plus Protein Dual Color protein ladder standard (Bio-Rad Laboratories, USA). The self-made gels were run at 160 V for 55 min with the PageRuler Plus Prestained protein ladder (Bio-Rad Laboratories, USA).

### Evaluation of data

We first evaluated the reproducibility of the reference runs by calculating the absolute average error (Θ) (Eq. 2).

2 $$\Theta \left[ \% \right] = \left( {\frac{{\mathop \sum \nolimits_{i = 1}^{n} \left| {\overline{x}_{ave} - \overline{x}_{i} } \right|}}{n}} \right){ } \times \frac{100}{{\overline{x}_{ave} }}$$

${\stackrel{-}{x}}_{ave}$—average value of ${\stackrel{-}{x}}_{i}$ from all reference runs; ${\stackrel{-}{x}}_{i}$—calculated average of the respective parameter; *n*—number of cultivations (*n* = 3).

Then, the significance of the difference between the respective responses was evaluated by one-sample two-tailed *t* test. In this test, a population (the three reference runs) was examined whether it was significantly different from the deviation run. For that purpose, a null hypothesis (H_0_) was defined: “The value of a parameter of the deviation run is equal to the average value of the reference runs”. The alternative hypothesis (H_1_) was defined as: “The value of a parameter of the deviation run is not equal to the average value of the reference runs”. If the absolute calculated value of t (Eq. 3) was higher than the *t* value t_α_, derived from a student’s t distribution table, H_0_ was rejected:

3 $$t = \frac{{\overline{{x_{0} }} - x_{1} }}{{\frac{\sigma }{\sqrt n }}}$$

$\stackrel{-}{{x}_{0}}$—average value of reference runs; ${x}_{1}$—value of deviation run; σ—standard deviation of reference runs; *n*—number of measurements.

All data were compared by a confidence interval of 95% (α = 0.05) and 99% (α = 0.01). In the USP, we investigated and compared growth, substrate concentrations, metabolite concentrations, physiological yields and target protein production for samples after the deviation phase and at cultivation end. In the DSP, the critical quality attributes (CQA) of the IMAC-purified target proteins were assessed (Table 4). During size-exclusion (SEC) HPLC, the fluorescence was measured for GFP with a FLD-3400RS module (ThermoFisher Scientific, Austria). A SEC HPLC column from Waters GmbH (Austria) was used (XBridge® BEH200A SEC 3.5 µm). The Reinheitszahl (RZ) was determined with a NanoDrop 1000 spectrophotometer (Thermo Scientific, Austria).

**Table 4:** Overview of protein quality attributes and the respective analysis methods

	CQA	GFP	P2Ox
Protein variations	Homogeneity in hydrophobicity [%]	RP HPLC at 280 nm	RP HPLC at 395 nm
Protein size	Homogeneity in size [%]	SEC HPLC with excitation 491 nm and emission 512 nm	SEC HPLC at 395 nm
Protein activity	Specific activity [−] or [U mg^−1^]	SEC HPLC with excitation 491 nm and emission 512 nm versus absorbance at 280 nm	ABTS and BCA assay
Cofactor loading	Reinheitszahl [−]	–	420 nm versus 280 nm

## Results and discussion

### Generation and characterization of GFP and P2Ox standards

**Fig. 1:**
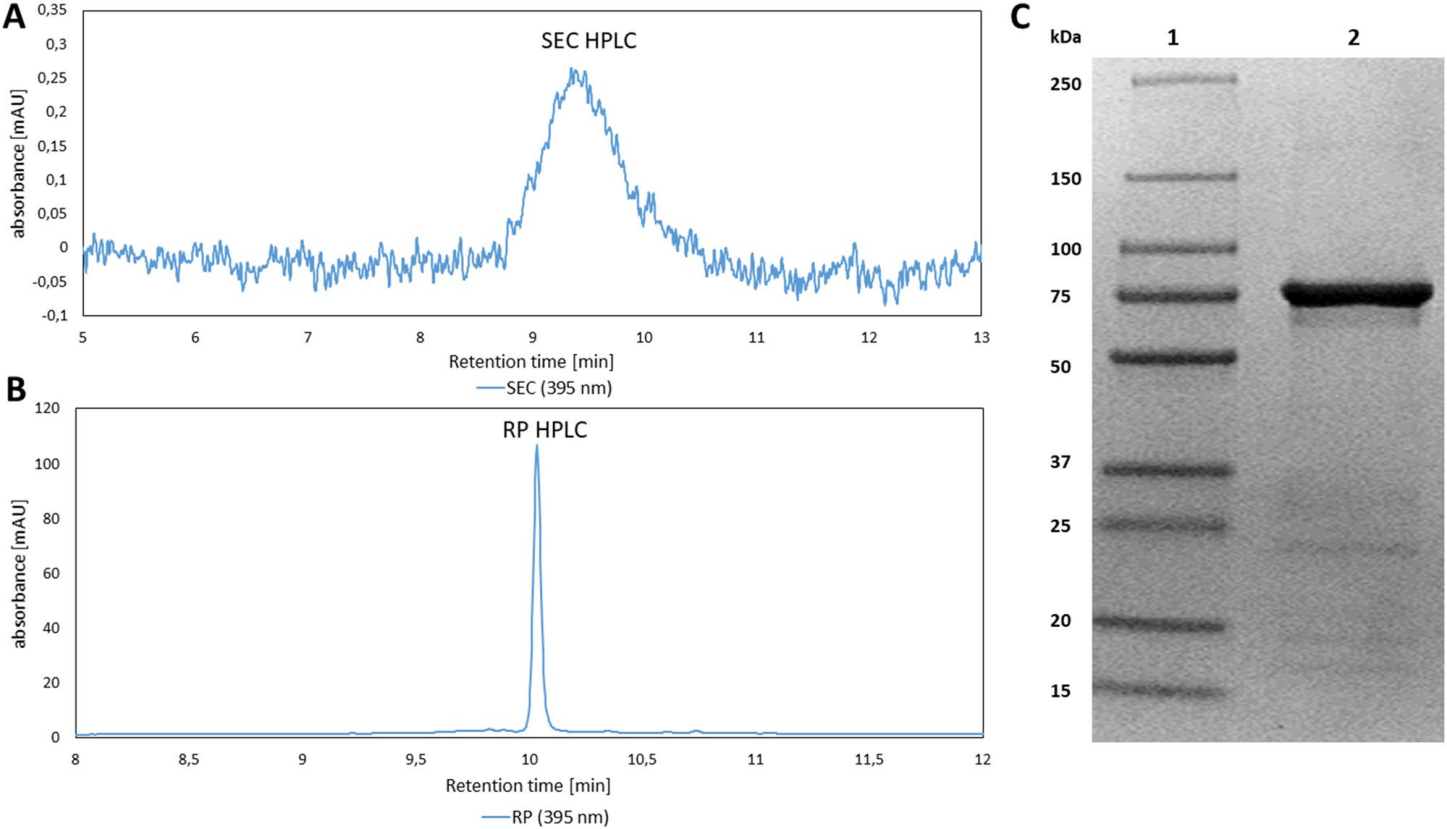
P2Ox protein standard quality analysis. **a** SEC HPLC with absorption at 395 nm, characteristic for P2Ox with bound FAD. **b** RP HPLC with absorption at 395 nm, characteristic for P2Ox with bound FAD. **c** 4–15% gradient SDS-PAGE. Lane 1 contains the ladder, lane 2 the IMAC purified P2Ox standard with a clear band around 75 kDa that represents the denatured P2Ox

**Fig. 2:**
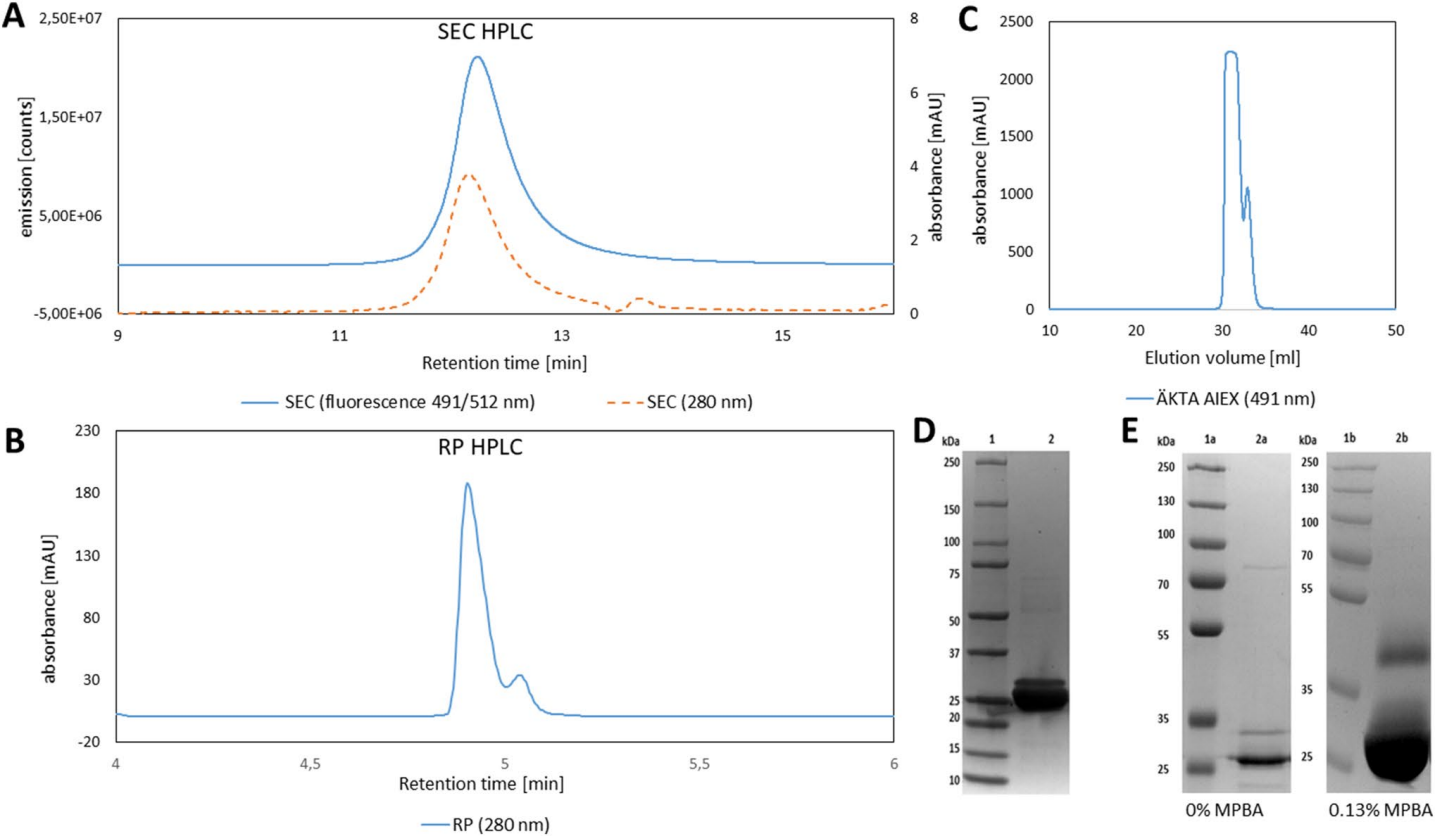
GFP protein standard quality analysis. **a** SEC HPLC with absorption at 280 nm (−) and fluorescence with excitation at 491 nm and emission at 512 nm (- -), characteristic for GFP. **b** RP HPLC with absorption at 280 nm with visible double peak pattern. **c** Äkta AIEX chromatogram of GFP at 491 nm absorption. A second peak is seen for the IMAC purified GFP standard, which eluted at higher salt concentrations. **d** 4–15% gradient SDS-PAGE. Lane 1 contains the ladder, lane 2 the IMAC purified GFP standard with a clear band around 25 kDa and a higher band around 30 kDa. **e** Self-cast SDS-PAGE gels for protein glycation analysis. left: Gel without MPBA; lane 1a shows the protein ladder; lane 2a shows the IMAC purified GFP standard with two bands. Right: Gel with 0.13% MPBA; lane 1b shows the protein ladder; lane 2b shows the IMAC purified GFP standard with a band around 25 kDa and a clearly elevated second band at around 45 kDa for the presumably glycated GFP species

The P2Ox standard was of high homogeneity and purity after IMAC purification (Fig. 1). In contrast, the GFP standard showed a distinct second, more hydrophobic peak in the RP HPLC analysis (Fig. 2b). Interestingly, this second peak was not visible in SEC HPLC (Fig. 2a), but a second distinct band was clearly visible above the expected GFP band (~ 28 kDa) in SDS-PAGE analysis (Fig. 2d). We hypothesized this protein to be a modified GFP species with an intact his-tag that was co-purified by IMAC. Thus, we analysed the GFP standard further by AIEX at 491 nm. As we hypothesized, the double peak pattern from RP HPLC and SDS-PAGE analysis was also visible in AIEX (Fig. 2c). We concluded that a fraction of soluble GFP underwent intracellular protein modification. However, *E. coli* has only limited capacities for post-translational protein modifications. Therefore, modified GFP could result from non-enzymatic modifications, like protein glycation. Although the used GFP construct contained no N-terminal his-tag, glycation might have happened [14].

Geoghegan et al*.* showed that protein modification with 6-PGL or its dephosphorylated version results in a more hydrophobic protein character in RP HPLC [11]. In fact, we found this behavior (Fig. 2b). Thus, we employed a specifically developed SDS-PAGE to investigate potential GFP glycation [23]. When we compared band retention of produced GFP standard by normal SDS-PAGE and MPBA-containing SDS-PAGE (Fig. 2e), we noticed the appearance of a distinct band above 35 kDa in the MPBA-containing gel, underlining the probability of glycation. Although LC–MS/MS would be the ideal method to verify protein glycation, we concluded that glycation was likely present for a fraction of GFP based on our analyses.

### Reproducibility

Bioprocesses are generally regarded as reproducible, due to the controlled environment of a bioreactor and calculation of rates, yields and C-balances. However, sample handling and processing are error-prone procedures due to human interaction. Therefore, we performed three reference runs (C1–C3) to understand the inherent process variability. The average absolute error (Θ) of the cultivation end samples made it possible to properly understand the process variance (Supplementary Table 1). Generally, most variables showed good reproducibility with a Θ < 10%. However, protein assay-based variables showed an elevated Θ of around 20%. This variability was attributed to increased operator handling during these measurements.

### Impact of technical failures on the production and quality of a simple fold protein

In cultivation **C4-GFP** the aeration was interrupted, which might happen through blocked air filters, and introduced microaerobic conditions. In the deviation phase, an expected decrease in µ and Y_X/S_ to 0.02 h^−1^ and 0.13 Cmol Cmol^−1^ was found, respectively, but not in q_S, Glc_ (Table 5). Acetate and formate accumulation further highlighted that glucose was mainly used for mixed-acid fermentation. Due to decreased activity of the electron transport system (less ATP generation / increased NADH accumulation) under oxygen-limited conditions [24], redox balance is maintained by the generation of various organic acids [25]. Additionally, GFP productivity stagnated under these microaerobic conditions (Table 5). Secretion of organic acids led to strong pH fluctuations, base consumption (Supplementary Figure 4), low glucose accumulation, but no measureable degree of cell lysis. In the regeneration phase, GFP production was re-instated, glucose and acetate were consumed, but substrate to biomass conversion was clearly diminished, as Y_X/S_ remained low at 0.19 Cmol Cmol^−1^. Interestingly, we found a positive impact of this technical failure on GFP quality. The purified protein showed an increased homogeneity in hydrophobicity of 91.0%, compared to 85.9% in the reference runs (Table 6). We hypothesized reduced protein glycation to be the reason. Given that BL21(DE3) naturally has an impaired PPP due to missing PGL [13] and requires a bypass to produce ribulose-5-phosphate [24], a high flux through glycolysis and TCA would decrease the formation of free 6-PGL under microaerobic conditions [24].

**Table 5:** Phase specific influence of technical failures on the production process and the quantity of GFP

Sampling point = after technical failure							
	Parameter	$\overline{{\text{X}}}$± s (3 × ref.)	Interruption of aeration (C4-GFP)	Interruption of feeding (C5-GFP)	Failure in T control (C6-GFP)	Overfeeding (C7-GFP)	Failure in pH control (C8-GFP)
Growth and substrate consumption	q_S, Glc_ [g g^−1^ h^−1^]	0.13 ± 0.01	No	n.a	No	Yes ↑↑ 0.21 ± 0.01	No
	µ [h^−1^]	0.05 ± 0.01	Yes ↓ 0.02 ± 0.00	Yes ↓ − 0.01 ± 0.00	No	Yes ↑ 0.10 ± 0.00	No
Specific metabolite rates	q_Formate_ [mg g^−1^ h^−1^]	1.4 ± 3.8	Yes ↑↑ 55.3 ± 0.9	No	No	No	No
	q_Acetate_ [mg g^−1^ h^−1^]	0.3 ± 2.0	Yes ↑↑ 39.4 ± 0.7	Yes ↓ − 5.3 ± 0.1	No	No	No
Specific GFP rates and concentrations	q_GFP, int_ [mg g^−1^ h^−1^]	10.3 ± 1.8	Yes ↓↓ − 12.1 ± 1.5	Yes ↓↓ − 7.5 ± 0.9	Yes ↑↑ 34.1 ± 4.3	No	No
	c_GFP, ext,_ [mg g^−1^]	0.4 ± 0.4	No	No	No	No	No
	c_GFP, int_ [mg g^−1^]	75.5 ± 6.5	No	No	Yes ↑↑ 122.6 ± 1.3	No	No
Yields	Y_X/S_ [Cmol Cmol^−1^]	0.43 ± 0.05	Yes ↓ 0.13 ± 0.00	n.a	No	No	No
	Y _CO2/S_ [Cmol Cmol^−1^]	0.46 ± 0.03	n.a	n.a	No	Yes ↑ 0.60 ± 0.01	No
	Y _GFP, int/S_ [Cmol Cmol^−1^]	0.10 ± 0.01	Yes ↓↓ − 0.11 ± 0.01	n.a	Yes ↑↑ 0.34 ± 0.04	No	Yes ↓ 0.07 ± 0.01
Sampling point = after regeneration (cultivation end)							
Growth and substrate consumption	q_S,Glc_ [g g^−1^ h^−1^]	0.12 ± 0.01	Yes ↑ 0.17 ± 0.01	No	No	No	No
	µ [h^−1^]	0.04 ± 0.00	No	Yes ↑ 0.05 ± 0.00	No	No	No
Specific metabolite rates	q_Formate_ [mg g^−1^ h^−1^]	0.0 ± 0.6	Yes ↓ − 1.8 ± 0.1	Yes ↑ 1.8 ± 0.0	No	No	No
	q_Acetate_ [mg g^−1^ h^−1^]	0.0 ± 0.2	Yes ↓↓ − 12.4 ± 0.4	No	No	No	No
Specific GFP rates and IB formation	q_GFP, int_ [mg g^−1^ h^−1^]	12.5 ± 3.8	No	No	Yes ↑ 22.8 ± 1.3	No	No
	c_GFP, ext,_ [mg g^−1^]	0.6 ± 0.2	No	No	No	No	No
	c_GFP, int_ [mg g^−1^]	115.9 ± 11.0	No	No	Yes ↑↑ 195.9 ± 1.3	Yes ↑ 157.9 ± 1.3	No
	c_GFP, IB_ [mg g^−1^]	0.28 ± 0.02	No	No	No	No	No
Yields	Y_X/S_ [Cmol Cmol^−1^]	0.35 ± 0.04	Yes ↓ 0.19 ± 0.01	No	No	No	No
	Y _CO2/S_ [Cmol Cmol^−1^]	0.47 ± 0.04	No	No	No	No	No
	Y _GFP, int/S_ [Cmol Cmol^−1^]	0.12 ± 0.03	No	No	No	Yes ↑ 0.24 ± 0.02	No

Values were compared to the average values generated in three reference runs (3 × ref.). Statistical evaluation was done by one-sample two-tailed *t* test. Statistically relevant deviations are marked with “Yes” and arrows indicate the direction and magnitude of the deviation (α = 0.01 = ↑↑ or ↓↓ α = 0.05 = ↑ or ↓); “No” indicates that no significant deviation was found and “n.a.” indicates that comparison was not applicable in this case

**Table 6:** Influence of technical failures on GFP protein quality

Protein quality						
CQA	$\overline{{\text{X}}}$± s (3 × ref.)	Interruption of aeration (C4-GFP)	Interruption of feeding (C5-GFP)	Failure in T control (C6-GFP)	Overfeeding (C7-GFP)	Failure in pH control (C8-GFP)
Homogeneity in hydrophobicity [%]	85.9 ± 0.3	Yes ↑↑ 91.0	Yes ↑ 87.4	No	Yes ↑ 87.1	Yes ↑ 87.1
Homogeneity in size [%]	100 ± 0	No	No	No	No	No
Specific activity [−]	6.2 × 10^6^ ± 6.5 × 10^4^	No	No	Yes ↓ 6.0 × 10^6^	No	Yes ↑ 6.4 × 10^6^

Values were compared to the average value generated in three reference runs (3 × ref.). Statistical evaluation was done by one-sample two-tailed *t* test. Statistically relevant deviations are marked with “Yes” and arrows indicate the direction and magnitude of the deviation (α = 0.01 = ↑↑ or ↓↓ α = 0.05 = ↑ or ↓); “No” indicates that no significant deviation was found

In cultivation **C5-GFP**, we interrupted substrate addition. As expected CO_2_ evolution declined, DO increased and pH increased slowly due to uptake of organic acids (Supplementary Figutr 5). Glucose starvation induces ribosomal RNA (rRNA) degradation (~ 20% reduction of 16S rRNA after 1.3 h) [26] and accumulation of cyclic-AMP (cAMP) that activates catabolic promoters and inhibits anabolic promoters to maintain homeostatic energy levels [27]. Consequently, we found that biomass growth and GFP productivity stagnated (Table 5), however, cells did not lyse. Upon addition of glucose to starved cells, rRNA degradation [26] and intracellular cAMP concentrations [28] rapidly decrease. Therefore, growth and protein production should be re-instated, which was in accordance to our results that showed no altered physiology and GFP productivity with re-instated feeding at cultivation end (Table 5). GFP homogeneity increased slightly to 87.4% (Table 6).

The failure in temperature control in cultivation **C6-GFP** caused an increase in temperature from 27.5 °C to 33.5 °C at a rate of 4.0 °C h^−1^ during the deviation phase (Supplementary Figure 6). It was reported that a temperature upshift increases TCA flux, CO_2_ formation and heat-shock protein expression together with a reduction in growth physiology and biosynthesis of amino acids [29]. Interestingly researchers also showed that intracellular protein degradation and ribosomal peptide elongation rate increase with temperature [30]. Furthermore, T7 RNA polymerase, which is present in *E. coli* BL21(DE3), shows increased activity with temperature [31]. Together, this should yield an increased target protein titer in BL21(DE3) strains, given that the target protein is stable and does not easily form IBs. In fact, our results confirmed that as GFP production was boosted during the deviation phase (Table 5). The Y_GFP, int/S_ increased approximately threefold to 0.34 Cmol Cmol^−1^ compared to 0.10 Cmol Cmol^−1^ in the reference runs. However, we found no significant changes in growth physiology and CO_2_ formation. This likely resulted from the rather moderate increase in temperature compared to harsh shifts from 30 °C to 42 °C [29]. When we reinstated temperature control, a difference in Y_GFP,int/S_ was not present after the regeneration phase, however the final intracellular GFP concentration almost doubled to 195.9 ± 1.3 mg g^−1^ (Table 5). IB formation did not increase. This technical failure did not yield an impact on final GFP homogeneity, but a small decrease in specific GFP activity (Table 6). Absorption spectra of GFP are highly dependent on the protein’s amino acid composition and structure [32]. It is likely that increased GFP expression resulted in a small fraction of partially misfolded, but soluble GFP species with decreased activity.

In cultivation **C7-GFP**, we simulated overfeeding for 1.5 h. Glucose did not accumulate, although the pump setpoint was set to its maximum. We found a twofold increased µ and q_S,Glc_ in the deviation phase (Table 5). Interestingly, an increased Y_CO2/S_ of 0.60 Cmol Cmol^−1^ was also present in the deviation phase. BL21-based strains naturally have a high flux through the glyoxylate shunt, due to low expression of IclR (isocitrate lyase repressor) [33, 34] and, therefore, the high activity of isocitrate lyase (isocitrate glyoxylate + succinate [35]). Interestingly, experiments under glucose-limited conditions showed slightly increasing amounts of IclR in *E. coli* BL21(DE3) over time [36]. Therefore, it might be possible that a certain amount of IclR was present before the technical failure. Then, a sudden increase in glucose feeding could have increased flux through CO_2_ generating reactions of the TCA (isocitrate α-ketoglutarate succinyl-CoA [35]) without increasing flux through the glyoxylate shunt. This might explain the increased Y_CO2/S_. At cultivation end, we surprisingly found a twofold increase in Y_GFP, int/S_ and around 40% more GFP per biomass (Table 5). We further found a slightly elevated homogeneity of the purified GFP to 87.1% (Table 6).

In cultivation **C8-GFP**, we stopped base addition for 1.5 h to simulate a defect base pump. Consequently, the pH decreased to pH 6.4 by 0.35 h^−1^ in the deviation phase (Supplementary Figure 8). Interestingly, we detected a small decrease of Y_GFP/S, int_ in the deviation phase (Table 5). Cytoplasmic pH is tightly regulated [37], however, the acidifying extracellular environment could have caused intracellular stress that led to decreased GFP expression. Importantly, no physiological and productivity-related differences were found at cultivation end. Again, we found a slightly increased homogeneity of hydrophobicity to 87.1%, but also a small increase in GFP specific activity (Table 6).

### Impact of technical failures on the production and quality of a protein with complex fold

In **C4-P2Ox**, we found an increase in metabolite formation during the deviation phase, but no other enduring change (Table 7).

**Table 7:** Phase specific influence of technical failures on the production process and the quantity of P2Ox

Sampling point = after technical failure							
	Parameter	$\overline{{\text{X}}}$± s (3 × ref.)	Interruption of aeration (C4-P2Ox)	Interruption of feeding (C5-P2Ox)	Failure in T control (C6-P2Ox)	Overfeeding (C7-P2Ox)	Failure in pH control (C8-P2Ox)
Growth and substrate consumption	q_S, Glc_ [g g^−1^ h^−1^]	0.08 ± 0.00	No	n.a	No	Yes ↑↑ 0.11 ± 0.02	No
	µ [h^−1^]	0.03 ± 0.00	Yes ↓ 0.01 ± 0.00	Yes ↓ 0.01 ± 0.00	Yes ↑ 0.04 ± 0.00	Yes ↑↑ 0.08 ± 0.00	No
Specific metabolite rates	q_Formate_ [mg g^−1^ h^−1^]	0.1 ± 0.2	Yes ↑↑ 34.0 ± 1.4	No	No	Yes ↑↑ 4.8 ± 0.2	No
	q_Acetate_ [mg g^−1^ h^−1^]	0.2 ± 0.9	Yes ↑↑ 29.4 ± 1.2	No	No	No	No
Specific P2Ox rates and concentrations	q_P2Ox, int_ [mg g^−1^ h^−1^]	2.77 ± 0.44	No	Yes ↓↓ − 0.54 ± 0.29	No	No	Yes ↓↓ − 1.36 ± 0.98
	c_P2Ox, ext_, [mg g^−1^]	0.00 ± 0.00	No	No	No	No	No
	c_P2Ox, int_ [mg g^−1^]	11.4 ± 2.4	No	No	No	No	Yes ↓ 5.2 ± 2.1
	act_P2Ox,int_ [U g^−1^]	38.0 ± 5.6	Yes ↓ 13.9 ± 1.6	Yes ↓ 22.8 ± 11.1	No	No	No
Yields	Y_X/S_ [Cmol Cmol^−1^]	0.41 ± 0.02	Yes ↓↓ 0.07 ± 0.00	n.a	No	No	No
	Y _CO2/S_ [Cmol Cmol^−1^]	0.49 ± 0.01	n.a	n.a	No	No	Yes ↑ 0.54 ± 0.00
	Y _P2Ox/S, int_ [Cmol Cmol^−1^]	0.04 ± 0.01	No	n.a	No	No	Yes ↓↓ − 0.02 ± 0.02
Sampling point = after regeneration (cultivation end)							
Growth and substrate consumption	q_S_ [g g^−1^ h^−1^]	0.08 ± 0.00	No	No	No	Yes ↑↑ 0.11 ± 0.01	No
	µ [h^−1^]	0.03 ± 0.00	No	No	No	Yes ↑ 0.04 ± 0.00	No
Specific metabolite rates	q_Formate_ [mg g^−1^ h^−1^]	0.5 ± 0.2	Yes ↓ − 0.1 ± 0.0	No	No	Yes ↓↓ − 0.7 ± 0.0	No
	q_Acetate_ [mg g^−1^ h^−1^]	0.2 ± 0.1	Yes ↓↓ − 9.1 ± 0.5	No	No	No	No
Specific P2Ox rates and concentrations	q_P2Ox, int_ [mg g^−1^ h^−1^]	6.52 ± 1.54	No	No	No	No	No
	c_P2Ox, ext,_ [mg g^−1^]	0.00 ± 0.00	No	No	No	No	No
	c_P2Ox, int_ [mg g^−1^]	37.3 ± 4.7	No	No	No	No	No
	act_P2Ox,int_ [U g^−1^]	158.6 ± 15.5	No	No	No	No	No
IB formation	c_P2Ox, IB_ [mg g^−1^]	3.4 ± 1.0	No	No	Yes ↑ 8.6 ± 0.2	No	No
Yields	Y_X/S_ [Cmol Cmol^−1^]	0.48 ± 0.05	No	No	No	No	No
	Y _CO2/S_ [Cmol Cmol^−1^]	0.49 ± 0.02	No	No	No	Yes ↑↑ 0.61 ± 0.00	No
	Y _P2Ox/S, int_ [Cmol Cmol^−1^]	0.11 ± 0.02	No	No	No	Yes ↓ 0.05 ± 0.01	No

Values were compared to the average value generated in three reference runs (3 × ref.). Statistical evaluation was done by one-sample two-tailed* t* test. Statistically relevant deviations are marked with “Yes” and arrows indicated the direction and magnitude of the deviation (α = 0.01 = ↑↑ or ↓↓ α = 0.05 = ↑ or ↓); “No” indicates that no significant deviation was found and “n.a.” indicates that comparison was not applicable in this case

In **C5-P2Ox**, growth and P2Ox productivity stagnated, similarly to C5-GFP (Table 7). When feeding was re-instated, cells showed no change in physiology nor P2Ox productivity.

In **C6-P2Ox**, the temperature increased from 20.0 °C to 27.7 °C with a rate of 5.2 °C h^−1^ (Supplementary Figure 14). Interestingly, this increase did not affect cell physiology nor productivity in the deviation phase (Table 7). Later analysis of biomass from cultivation end showed that P2Ox production was boosted, however, only in form of IBs. This is common for complex, recombinant proteins in *E. coli* and highlighted that P2Ox required gentle cultivation conditions to yield soluble protein.

In contrast to C7-GFP, increased feeding resulted in glucose accumulation to 3.5 g L^−1^ in **C7-P2Ox**. Acetate was not secreted due to generally low acetate production of BL21-based strains [24], but formate was produced [24]. When correct feeding was re-instated, an increased µ and q_S,Glc_ remained due to uptake of accumulated glucose. The amount of soluble P2Ox did not increase neither in a soluble form nor as IBs.

The failure in pH control in **C8-P2Ox** greatly affected protein production in the deviation phase (Table 7). Although P2Ox is a rather stable enzyme around pH 7.0 [38] and the pH decreased from pH 7.2 to only pH 6.9, this small extracellular pH shift resulted in a twofold decrease of intracellular P2Ox concentration.

Notably, a change in P2Ox quality was not detected by any technical failure (Table 8). This was probably linked to the low specific growth rate.

**Table 8:** Influence of technical failures on P2Ox protein quality

Protein quality						
Parameter	$\overline{{\text{X}}}$± s (3 × ref.)	Interruption of aeration (C4-P2Ox)	Interruption of feeding (C5-P2Ox)	Failure in T control (C6-P2Ox)	Overfeeding (C7-P2Ox)	Failure in pH control (C8-P2Ox)
Homogeneity in hydrophobicity [%]	100 ± 0.0	No				
Homogeneity in size [%]	100 ± 0.0					
Specific activity [U mg^−1^]	2.8 ± 1.0					
Reinheitszahl [−]	0.05 ± 0.01					

Values were compared to the average value generated in three reference runs (3 × ref.). Statistical evaluation was done by one-sample two-tailed t-test. Statistically relevant deviations are marked with “Yes” and arrows indicated the direction and magnitude of the deviation (α = 0.01 = ↑↑ or ↓↓ α = 0.05 = ↑ or ↓); “No” indicates that no significant deviation was found

## Conclusions

Recombinant protein production in *E. coli* BL21(DE3) is important in both academia and industry. In this follow-up study, we investigated the impact of common technical failures during recombinant production of two soluble, model proteins (GFP and P2Ox) on cell physiology, productivity and final protein quality. Our methodology also proved to be an interesting approach to investigate the process and product robustness. Compared to our previous results on IB production [3], our current results show that soluble protein production is less robust to temporary environmental changes. Importantly, we found that technical failures, especially those that introduce limited oxygen supply, significantly affect cell physiology, productivity, protein quality and, therefore, also final process and product robustness.

## Supplementary Information

Below is the link to the electronic supplementary material.

Supplementary file1 (DOCX 704 KB)

Supplementary file2 (DOCX 15 KB)

Supplementary file3 (XLSX 31 KB)
