# Supplementary material for: The impact of technical failures on recombinant production of soluble proteins in Escherichia coli: a case study on process and protein robustness
Source: Bioprocess Biosyst Eng. 2021 Jan 24;44(6):1049–61. doi: 10.1007/s00449-021-02514-w (PMC8144139; doi:10.1007/s00449-021-02514-w)
Supplement: Supplementary file 1 — Supplementary file1 (DOCX 704 KB) [file 449_2021_2514_MOESM1_ESM.docx]

**The impact of technical failures on recombinant production of soluble proteins in *Escherichia coli*: A case study on process and protein robustness**

Alexander Pekarsky^1^, Melanie Reninger^1^ and Oliver Spadiut^1*^

^1^ Technische Universität Wien, Institute of Chemical, Environmental and Bioscience Engineering, Research Area Biochemical Engineering, Gumpendorferstrasse 1a, 1060 Vienna, Austria

^*^ Correspondence: Oliver Spadiut, TU Wien, Institute of Chemical, Environmental and Bioscience Engineering, Research Area Biochemical Engineering, Gumpendorferstrasse 1a, 1060 Vienna, Austria. Tel: +43 1 58801 166473, Fax: +43 1 58801 166980, Email: oliver.spadiut@tuwien.ac.at

Alexander Pekarsky: alexander.pekarsky@tuwien.ac.at ; ORCID: 0000-0002-7330-9661

Melanie Reininger: melanie.reininger@students.boku.ac.at

Oliver Spadiut: oliver.spadiut@tuwien.ac.at ; ORCID: 0000-0003-0916-0644

**Keywords**

Process deviation, *Escherichia coli*, Cytosolic protein, Robustness, Bioreactor, Protein glycation


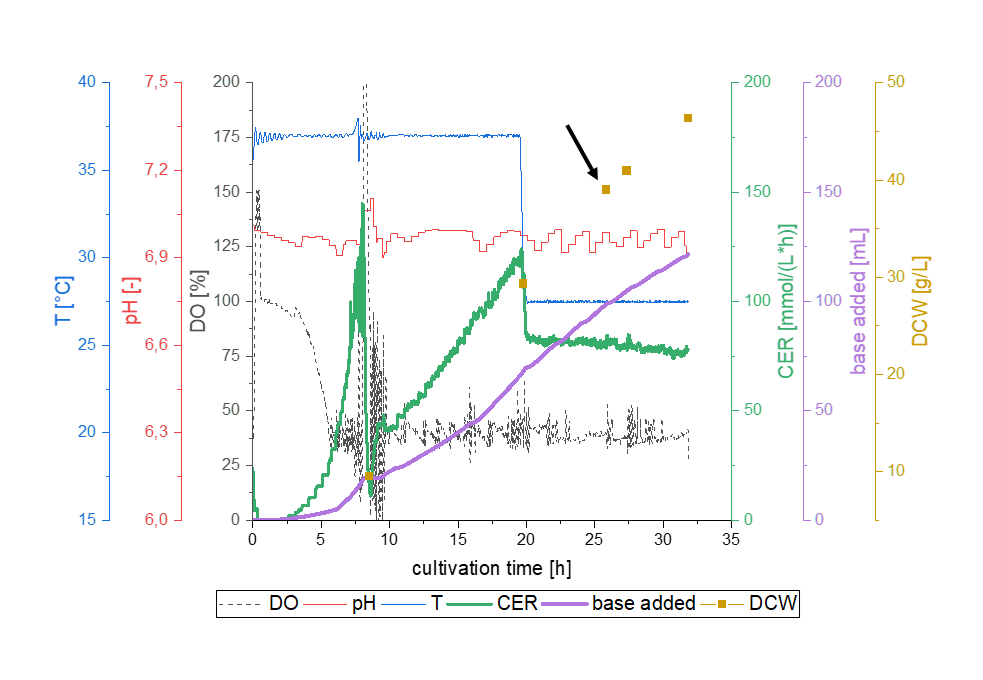


Figure 1: Process data for cultivation C1-GFP (reference run). Start of induced fed-batch is marked by drop in temperature. Process data of temperature (T), pH, dissolved oxygen (DO), added base, carbon dioxide evolution rate (CER) and dry cell weight (DCW) is shown for batch, uninduced fed-batch and induction phase.


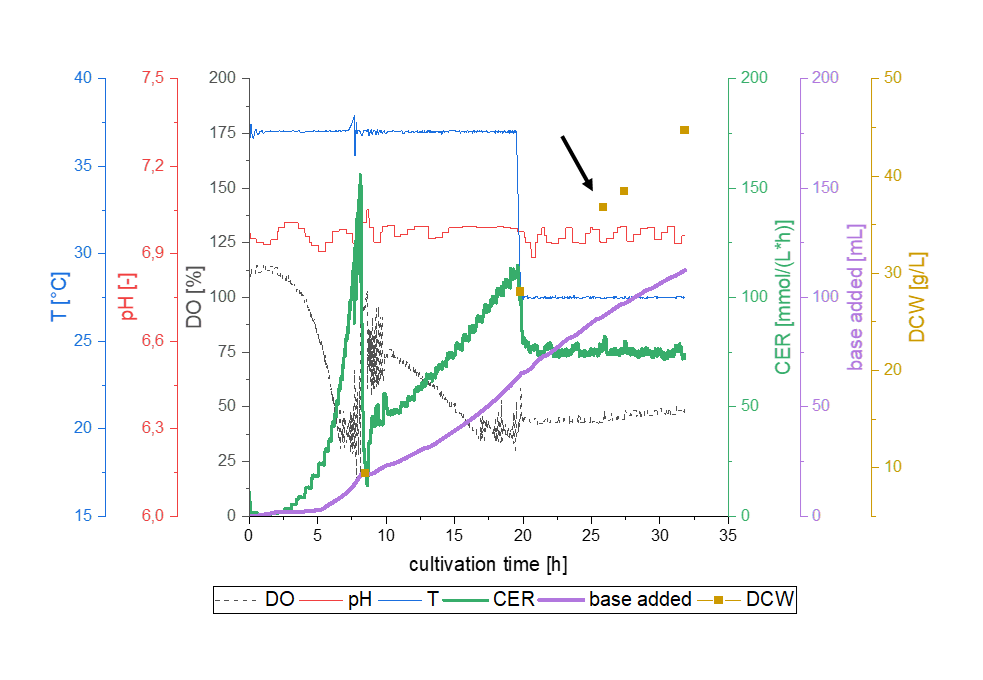


Figure 2: Process data for cultivation C2-GFP (reference run). Start of induced fed-batch is marked by drop in temperature. Process data of temperature (T), pH, dissolved oxygen (DO), added base, carbon dioxide evolution rate (CER) and dry cell weight (DCW) is shown for batch, uninduced fed-batch and induction phase.


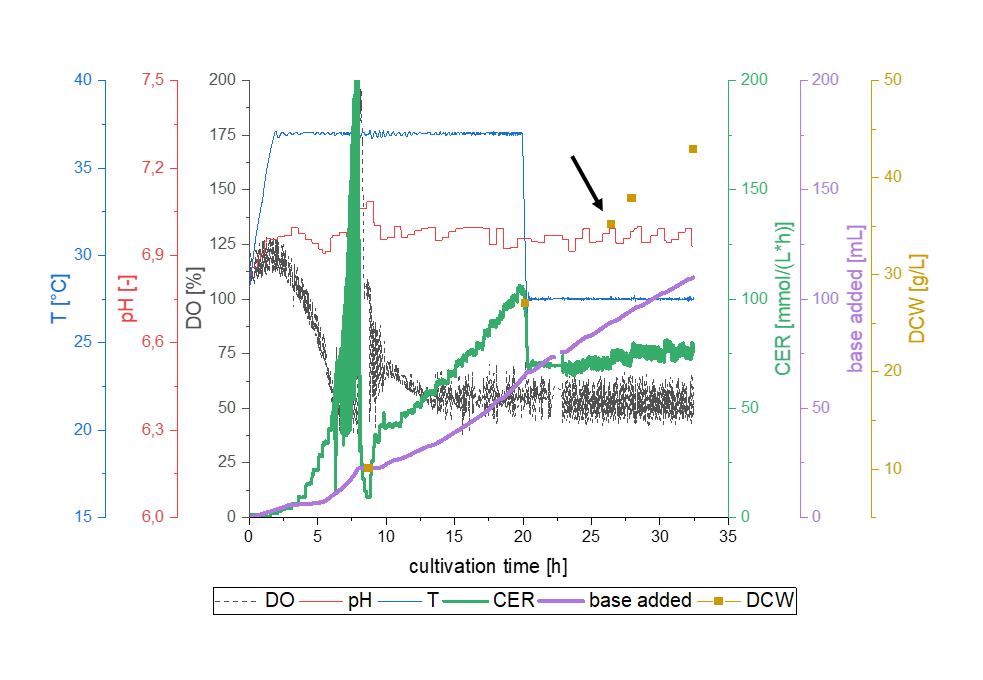


Figure 3: Process data for cultivation C3-GFP (reference run). Start of induced fed-batch is marked by drop in temperature. Process data of temperature (T), pH, dissolved oxygen (DO), added base, carbon dioxide evolution rate (CER) and dry cell weight (DCW) is shown for batch, uninduced fed-batch and induction phase.


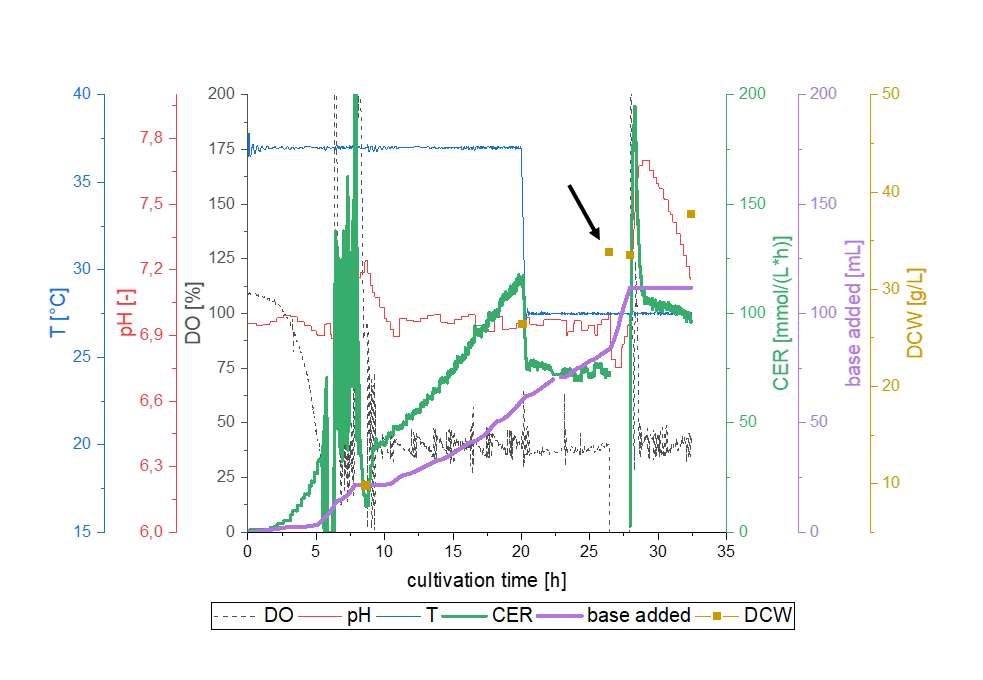


Figure 4: Process data for cultivation C4-GFP (interruption of aeration). Start of induced fed-batch is marked by drop in temperature, start of deviation through technical failure is marked by black arrow. Herein, the aeration was stopped for appr. 1.5 h, which was followed by a regeneration phase under standard conditions. Process data of temperature (T), pH, dissolved oxygen (DO), added base, carbon dioxide evolution rate (CER) and dry cell weight (DCW) is shown for batch, uninduced fed-batch and induction phase.


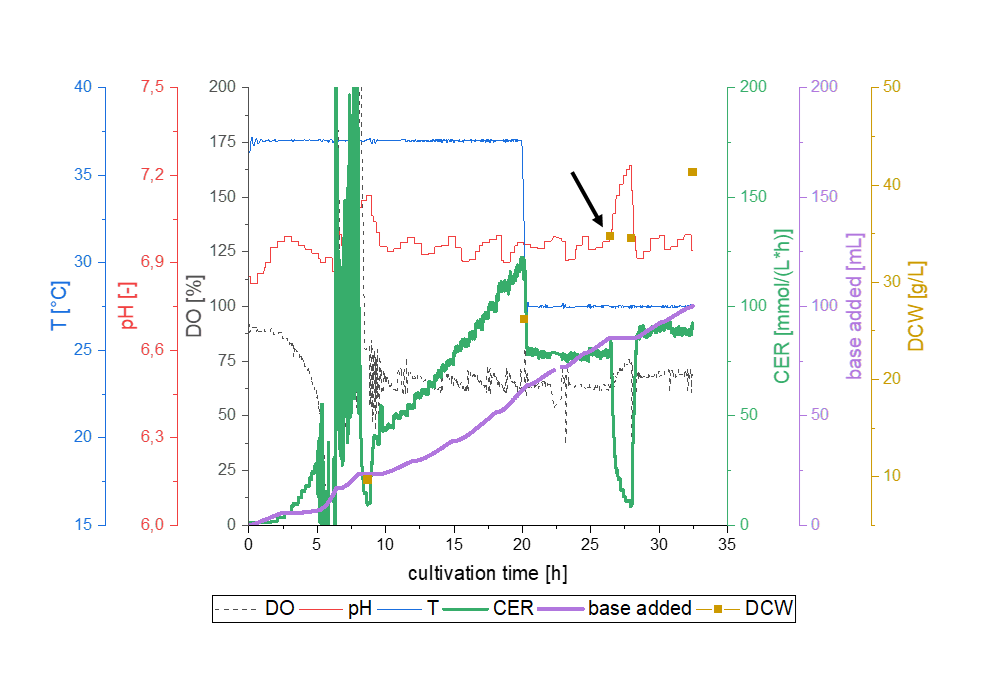


Figure 5: Process data for cultivation C5-GFP (interruption of feeding). Start of induced fed-batch is marked by drop in temperature, start of deviation through technical failure is marked by black arrow. Herein, the feeding was stopped for appr. 1.5 h, which was followed by a regeneration phase under standard conditions. Process data of temperature (T), pH, dissolved oxygen (DO), added base, carbon dioxide evolution rate (CER) and dry cell weight (DCW) is shown for batch, uninduced fed-batch and induction phase.


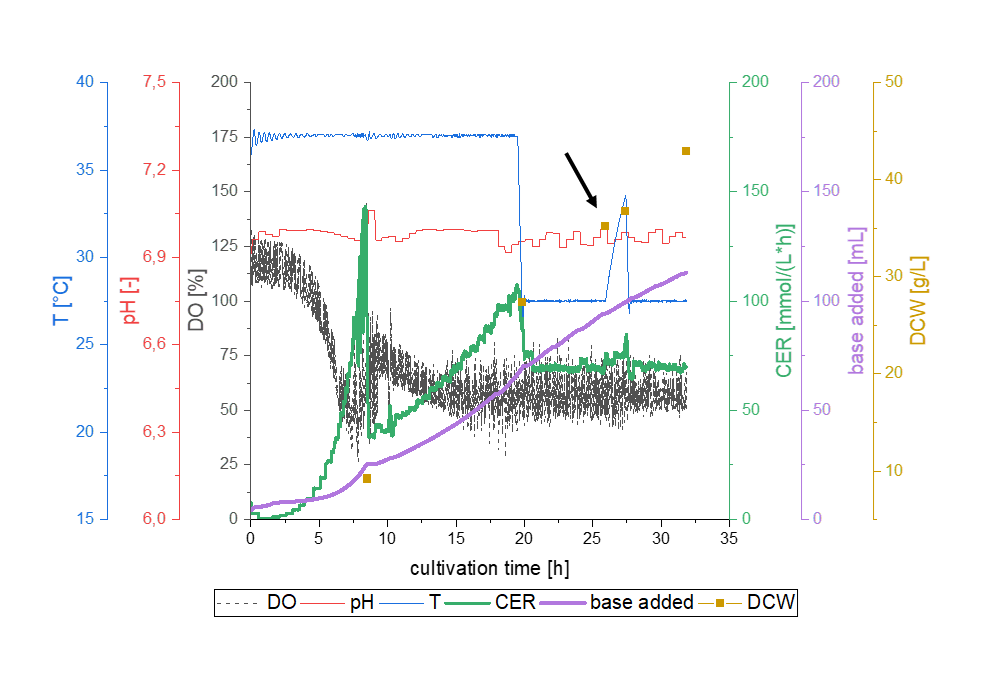


Figure 6: Process data for cultivation C6-GFP (failure in T control). Start of induced fed-batch is marked by drop in temperature, start of deviation through technical failure is marked by black arrow. Herein, the temperature control was stopped for appr. 1.5 h, which was followed by a regeneration phase under standard conditions. Process data of temperature (T), pH, dissolved oxygen (DO), added base, carbon dioxide evolution rate (CER) and dry cell weight (DCW) is shown for batch, uninduced fed-batch and induction phase.


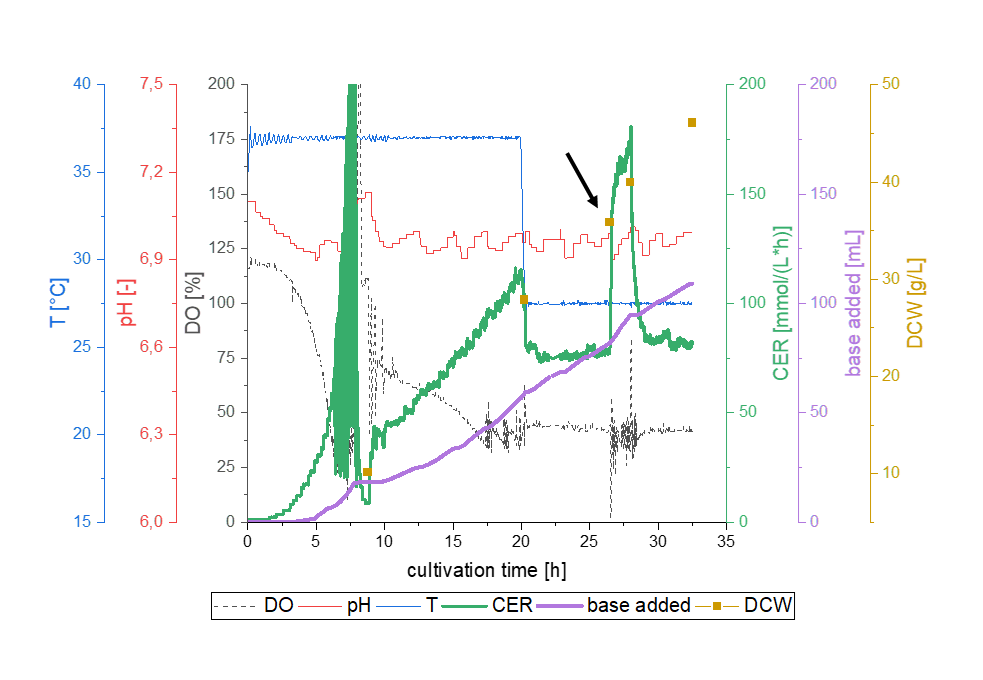


Figure 7: Process data for cultivation C7-GFP (overfeeding). Start of induced fed-batch is marked by drop in temperature, start of deviation through technical failure is marked by black arrow. Herein, the feed was increased for appr. 1.5 h, which was followed by a regeneration phase under standard conditions. Process data of temperature (T), pH, dissolved oxygen (DO), added base, carbon dioxide evolution rate (CER) and dry cell weight (DCW) is shown for batch, uninduced fed-batch and induction phase.


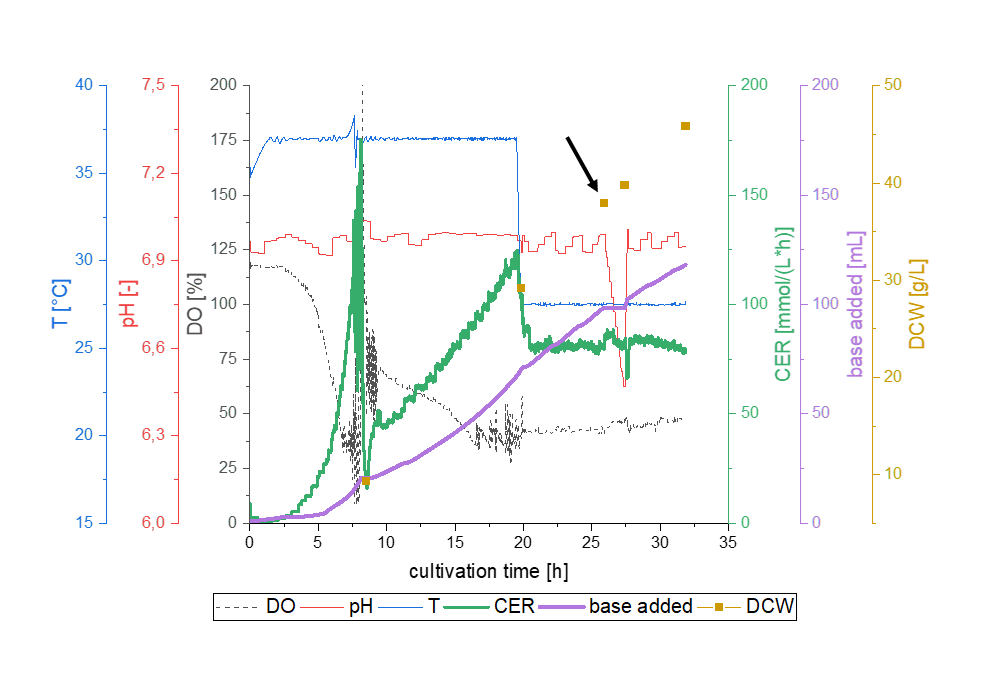


Figure 8: Process data for cultivation C8-GFP (failure in pH control). Start of induced fed-batch is marked by drop in temperature, start of deviation through technical failure is marked by black arrow. Herein, the pH control was stopped for appr. 1.5 h, which was followed by a regeneration phase under standard conditions. Process data of temperature (T), pH, dissolved oxygen (DO), added base, carbon dioxide evolution rate (CER) and dry cell weight (DCW) is shown for batch, uninduced fed-batch and induction phase.


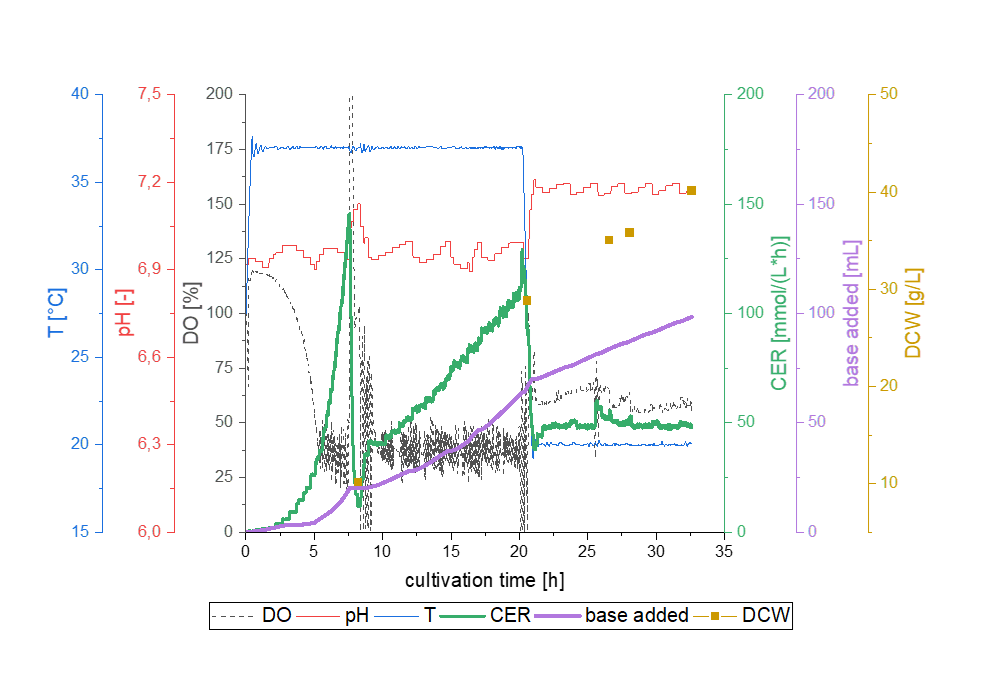


Figure 9: Process data for cultivation C1-P2Ox (reference run). Start of induced fed-batch is marked by drop in temperature. Process data of temperature (T), pH, dissolved oxygen (DO), added base, carbon dioxide evolution rate (CER) and dry cell weight (DCW) is shown for batch, uninduced fed-batch and induction phase.


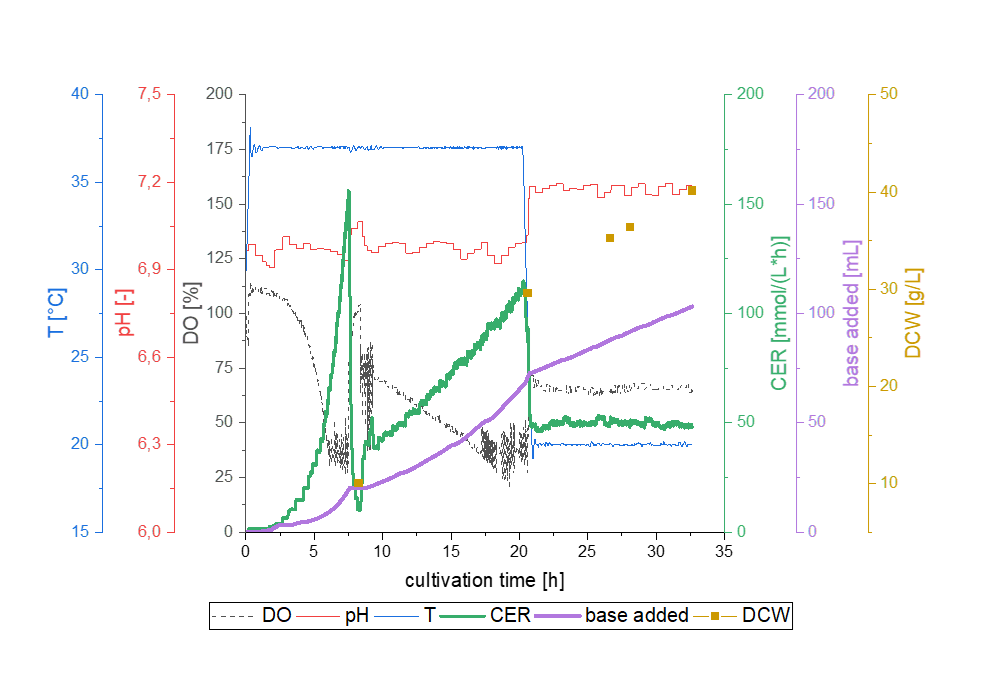


Figure 10: Process data for cultivation C2-P2Ox (reference run). Start of induced fed-batch is marked by drop in temperature. Process data of temperature (T), pH, dissolved oxygen (DO), added base, carbon dioxide evolution rate (CER) and dry cell weight (DCW) is shown for batch, uninduced fed-batch and induction phase.


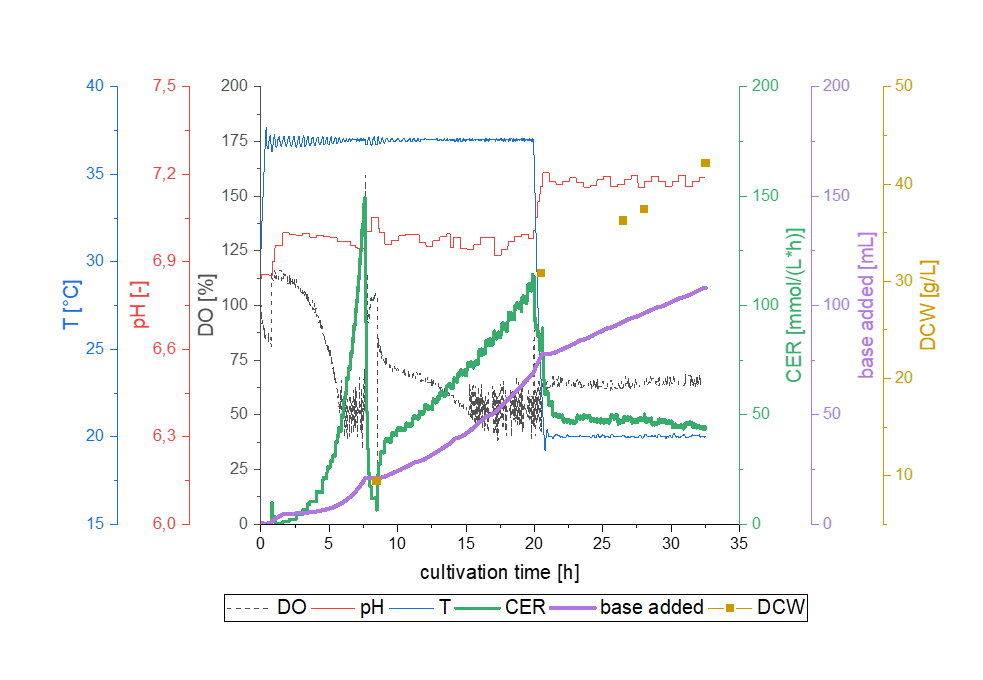


Figure 11: Process data for cultivation C3-P2Ox (reference run). Start of induced fed-batch is marked by drop in temperature. Process data of temperature (T), pH, dissolved oxygen (DO), added base, carbon dioxide evolution rate (CER) and dry cell weight (DCW) is shown for batch, uninduced fed-batch and induction phase.


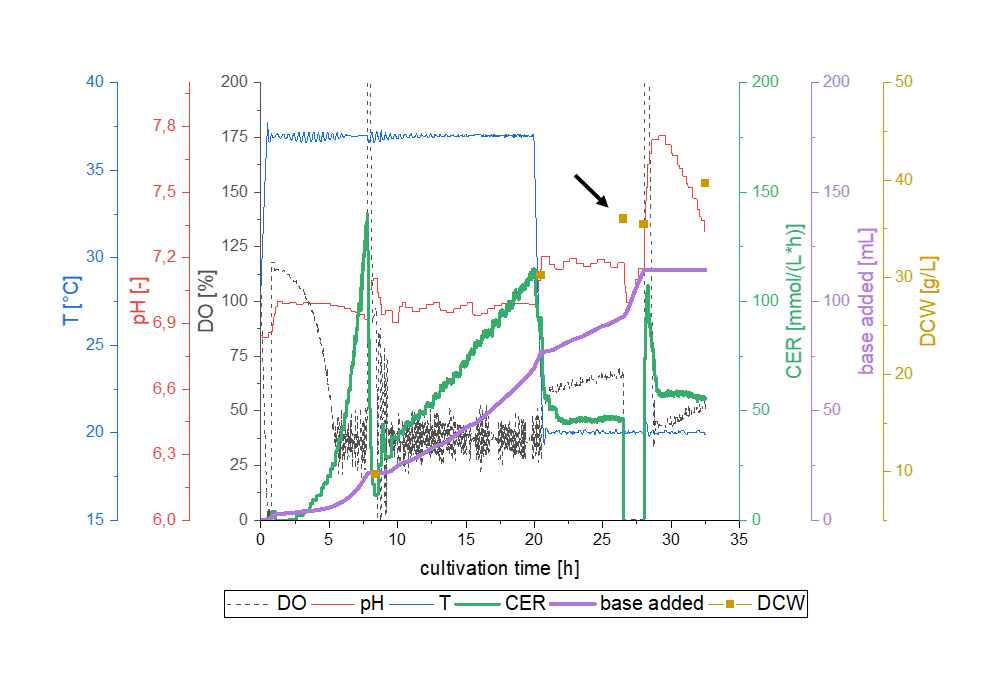


Figure 12: Process data for cultivation C4-P2Ox (interruption of aeration). Start of induced fed-batch is marked by drop in temperature, start of deviation through technical failure is marked by black arrow. Herein, the aeration was stopped for appr. 1.5 h, which was followed by a regeneration phase under standard conditions. Process data of temperature (T), pH, dissolved oxygen (DO), added base, carbon dioxide evolution rate (CER) and dry cell weight (DCW) is shown for batch, uninduced fed-batch and induction phase.


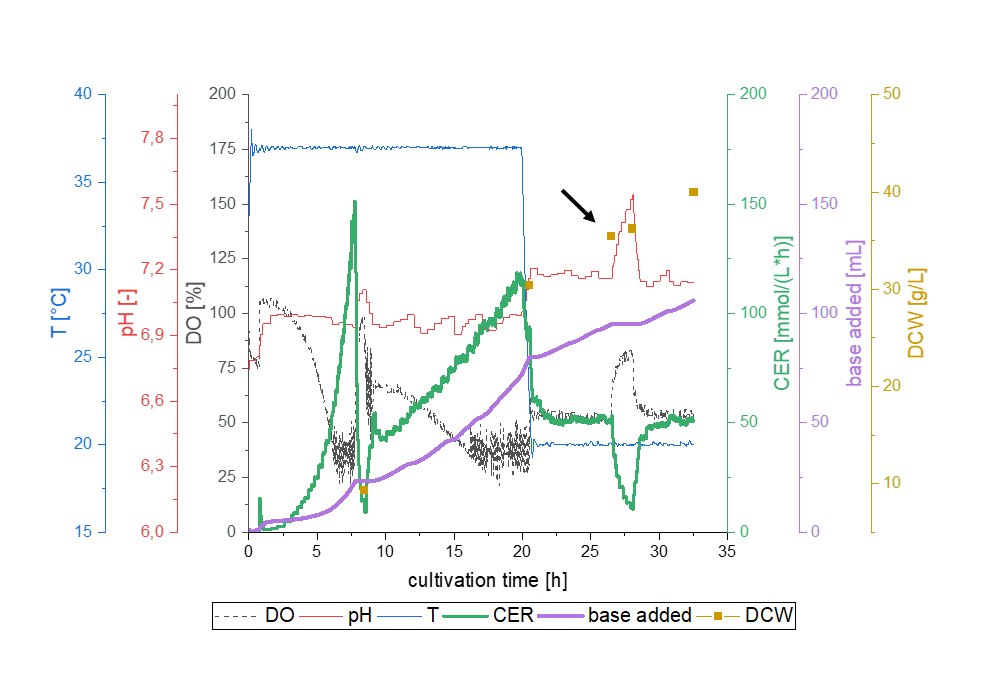


Figure 13: Process data for cultivation C5-P2Ox (interruption of feeding). Start of induced fed-batch is marked by drop in temperature, start of deviation through technical failure is marked by black arrow. Herein, the feeding was stopped for appr. 1.5 h, which was followed by a regeneration phase under standard conditions. Process data of temperature (T), pH, dissolved oxygen (DO), added base, carbon dioxide evolution rate (CER) and dry cell weight (DCW) is shown for batch, uninduced fed-batch and induction phase.


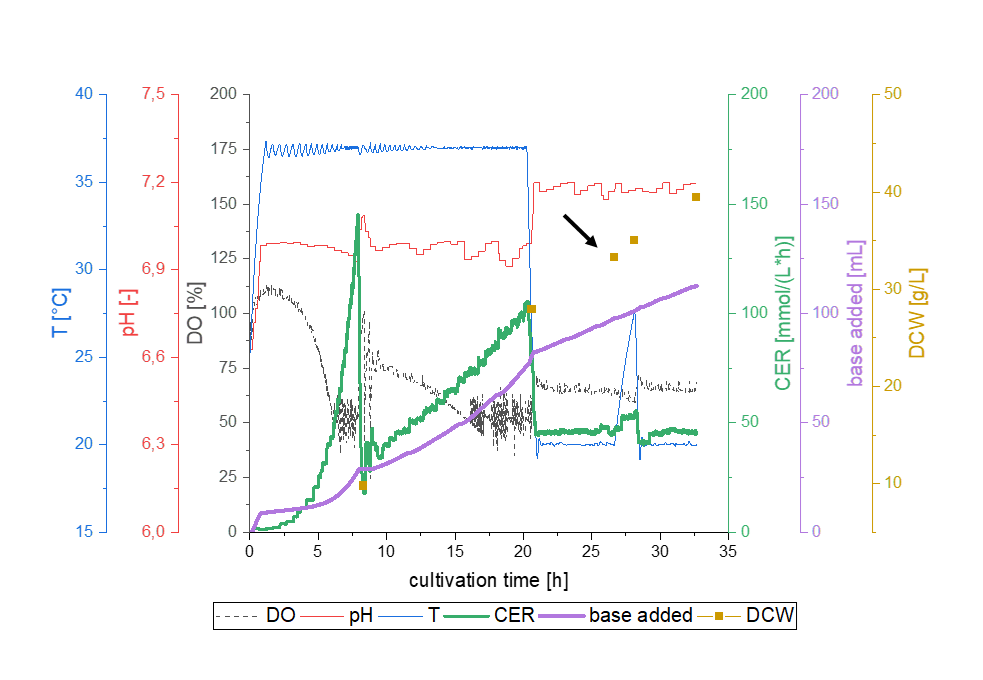


Figure 14: Process data for cultivation C6-P2Ox (failure in T control). Start of induced fed-batch is marked by drop in temperature, start of deviation through technical failure is marked by black arrow. Herein, the temperature control was stopped for appr. 1.5 h, which was followed by a regeneration phase under standard conditions. Process data of temperature (T), pH, dissolved oxygen (DO), added base, carbon dioxide evolution rate (CER) and dry cell weight (DCW) is shown for batch, uninduced fed-batch and induction phase.


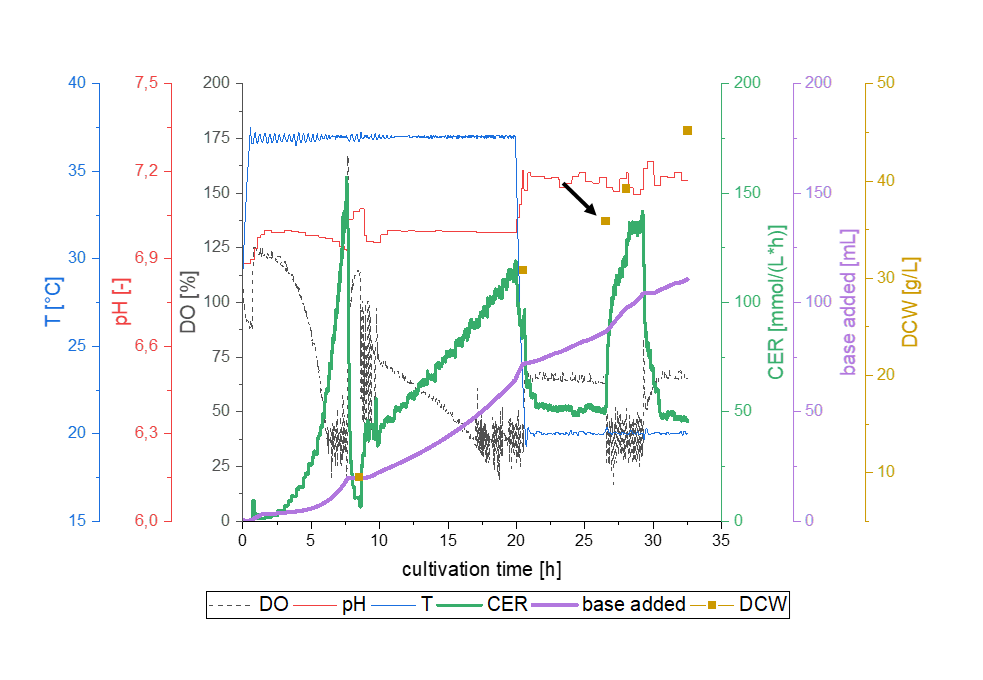


Figure 15: Process data for cultivation C7-P2Ox (overfeeding). Start of induced fed-batch is marked by drop in temperature, start of deviation through technical failure is marked by black arrow. Herein, the feed was increased for appr. 1.5 h, which was followed by a regeneration phase under standard conditions. Process data of temperature (T), pH, dissolved oxygen (DO), added base, carbon dioxide evolution rate (CER) and dry cell weight (DCW) is shown for batch, uninduced fed-batch and induction phase.


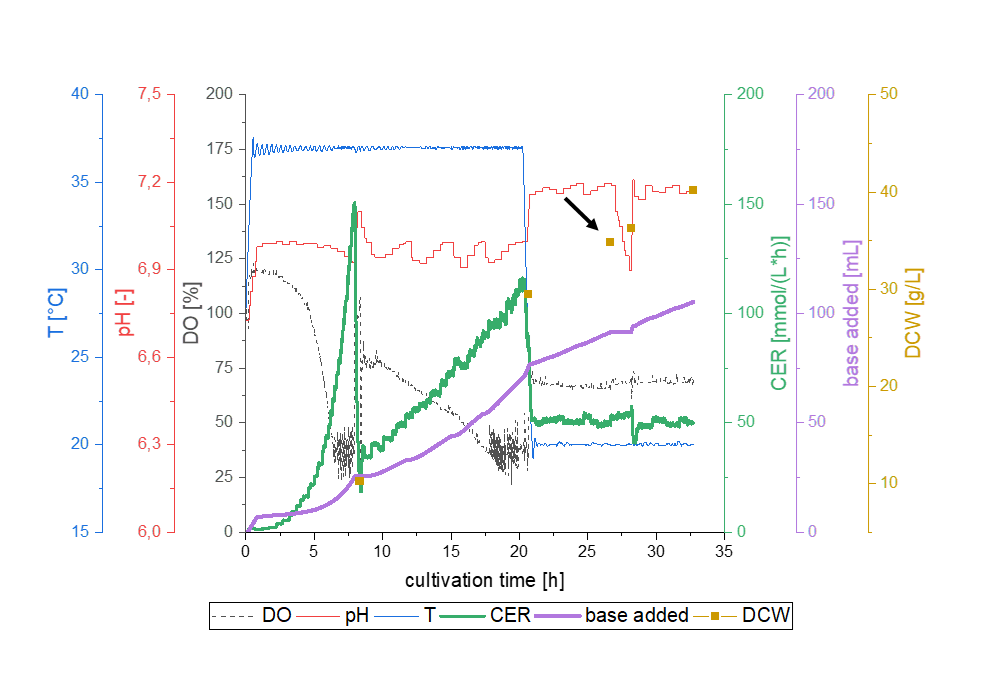


Figure 16: Process data for cultivation C8-P2Ox (failure in pH control). Start of induced fed-batch is marked by drop in temperature, start of deviation through technical failure is marked by black arrow. Herein, the pH control was stopped for appr. 1.5 h, which was followed by a regeneration phase under standard conditions. Process data of temperature (T), pH, dissolved oxygen (DO), added base, carbon dioxide evolution rate (CER) and dry cell weight (DCW) is shown for batch, uninduced fed-batch and induction phase.
