# Supplementary material for: The impact of technical failures on recombinant production of soluble proteins in Escherichia coli: a case study on process and protein robustness
Source: Bioprocess Biosyst Eng. 2021 Jan 24;44(6):1049–61. doi: 10.1007/s00449-021-02514-w (PMC8144139; doi:10.1007/s00449-021-02514-w)
Supplement: Supplementary file 2 — Supplementary file2 (DOCX 15 KB) [file 449_2021_2514_MOESM2_ESM.docx]

**The impact of technical failures on recombinant production of soluble proteins in *Escherichia coli*: A case study on process and protein robustness**

Alexander Pekarsky^1^, Melanie Reninger^1^ and Oliver Spadiut^1*^

^1^ Technische Universität Wien, Institute of Chemical, Environmental and Bioscience Engineering, Research Area Biochemical Engineering, Gumpendorferstrasse 1a, 1060 Vienna, Austria

^*^ Correspondence: Oliver Spadiut, TU Wien, Institute of Chemical, Environmental and Bioscience Engineering, Research Area Biochemical Engineering, Gumpendorferstrasse 1a, 1060 Vienna, Austria. Tel: +43 1 58801 166473, Fax: +43 1 58801 166980, Email: oliver.spadiut@tuwien.ac.at

Alexander Pekarsky: alexander.pekarsky@tuwien.ac.at ; ORCID: 0000-0002-7330-9661

Melanie Reininger: melanie.reininger@students.boku.ac.at

Oliver Spadiut: oliver.spadiut@tuwien.ac.at ; ORCID: 0000-0003-0916-0644

**Keywords**

Process deviation, *Escherichia coli*, Cytosolic protein, Robustness, Bioreactor, Protein glycation

Supplementary Table 1: Calculated average absolute error (Θ) for relevant upstream process and protein quality parameters for GFP and P2Ox at the end of cultivation.

| **Upstream process GFP+** | |
| --- | --- |
| **Parameter** | **Θ [%]** |
| DCW [g/L] | 2.6 |
| q_S_ [g/g/h] | 8.3 |
| µ [1/h] | 5.8 |
| q_Formate_ [mg/g/h] | 2159.5 |
| q_Acetate_ [mg/g/h] | 946 |
| q_GFP, int_ [mg/g/h] | 22.0 |
| c_GFP, ext,_[mg/g] | 19.9 |
| c_GFP, int_ [mg/g] | 7.3 |
| Y _X/S_ [C-mol/C-mol] | 8.0 |
| Y _CO2/S_ [C-mol/C-mol] | 5.8 |
| Y _GFP/S, int_ [C-mol/C-mol] | 17.2 |
| c_GFP, IB_ [mg/g] | 5.6 |
| **Protein quality GFP+** | |
| Homogeneity in hydrophobicity [%] | 0.2 |
| Homogeneity in size [%] | 0.0 |
| Specific activity [-] | 0.7 |
| **Upstream process P2Ox** | |
| DCW [g/L] | 2.2 |
| q_S_ [g/g/h] | 1.8 |
| µ [1/h] | 6.0 |
| q_Formate_ [mg/g/h] | 24.0 |
| q_Acetate_ [mg/g/h] | 28.3 |
| q_P2Ox, int_ [mg/g/h] | 16.3 |
| c_P2Ox, ext,_[mg/g] | 0.0 |
| c_P2Ox, int_ [mg/g] | 8.8 |
| Y _X/S_ [C-mol/C-mol] | 6.5 |
| Y _CO2/S_ [C-mol/C-mol] | 3.1 |
| Y _P2Ox/S, int_ [C-mol/C-mol] | 14.6 |
| c_P2Ox, IB_ [mg/g] | 21.5 |
| act_P2Ox_ [U/g] | 6.7 |
| **Protein quality P2Ox** | |
| Homogeneity in hydrophobicity [%] | 0.0 |
| Homogeneity in size [%] | 0.0 |
| Specific activity [U mg^-1^] | 25.6 |
| Reinheitszahl [-] | 14.0 |
